# Overexpression of Full-Length *ETV1* Transcripts in Clinical Prostate Cancer Due to Gene Translocation

**DOI:** 10.1371/journal.pone.0016332

**Published:** 2011-01-26

**Authors:** Delila Gasi, Hetty A. van der Korput, Hannie C. Douben, Annelies de Klein, Corrina M. de Ridder, Wytske M. van Weerden, Jan Trapman

**Affiliations:** 1 Department of Pathology, Erasmus University Medical Centre, Rotterdam, The Netherlands; 2 Department of Clinical Genetics, Erasmus University Medical Centre, Rotterdam, The Netherlands; 3 Department of Urology, Erasmus University Medical Centre, Rotterdam, The Netherlands; Southern Illinois University School of Medicine, United States of America

## Abstract

*ETV1* is overexpressed in a subset of clinical prostate cancers as a fusion transcript with many different partners. However, *ETV1* can also be overexpressed as a full-length transcript. Full-length ETV1 protein functions differently from truncated ETV1 produced by fusion genes. In this study we describe the genetic background of full-length *ETV1* overexpression and the biological properties of different full-length ETV1 isoforms in prostate cancer. Break-apart FISH showed in five out of six patient samples with overexpression of full-length *ETV1* a genomic rearrangement of the gene, indicating frequent translocation. We were able to study the rearrangements in more detail in two tumors. In the first tumor 5′-RACE on cDNA showed linkage of the complete *ETV1* transcript to the first exon of a prostate-specific two exon ncRNA gene that maps on chromosome 14 (*EST14*). This resulted in the expression of both full-length *ETV1* transcripts and *EST14-ETV1* fusion transcripts. In chromosome spreads of a xenograft derived from the second prostate cancer we observed a complex *ETV1* translocation involving a chromosome 7 fragment that harbors *ETV1* and fragments of chromosomes 4 and 10. Further studies revealed the overexpression of several different full-length transcripts, giving rise to four protein isoforms with different N-terminal regions. Even the shortest isoform synthesized by full-length *ETV1* stimulated *in vitro* anchorage-independent growth of PNT2C2 prostate cells. This contrasts the lack of activity of even shorter N-truncated ETV1 produced by fusion transcripts. Our findings that in clinical prostate cancer overexpression of full-length *ETV1* is due to genomic rearrangements involving different chromosomes and the identification of a shortened biologically active ETV1 isoform are highly relevant for understanding the mechanism of ETV1 function in prostate cancer.

## Introduction

Gene fusions are important in the development of many hematological malignancies and sarcomas, but are rare in most other tumor types [1]. However, the frequent fusion between *TMPRSS2* and the ETS gene *ERG* showed that gene fusion is a highly relevant event in prostate cancer [2], [3]. Overexpression of the *TMPRSS2-ERG* fusion gene has been reported in 40–70% of prostate cancer cases [2]–[5]. Fusions of *TMPRSS2* and three other genes encoding ETS transcription factors, *ETV1*, *ETV4* and *ETV5*, which are located on different chromosomes, occur at low frequency in prostate cancer [2], [6], [7]. However, for *ETV1* at least 10 different fusion partners have been described [8]–[10]. Most of these have in common that, like *TMPRSS2*, they are prostate-specific and androgen-regulated expressed. The properties of fusion partners are key elements in explaining the androgen-regulated overexpression of an ETS oncogene in prostate cancer. However, a unique characteristic of *ETV1* is that it can not only be overexpressed in prostate cancer as a fusion transcript but also as a full-length wild-type transcript.

The large family of ETS transcription factors is composed of 27 members [11]–[13]. All members have in common a highly homologous DNA binding domain, the ETS domain. The remaining regions of most ETS proteins show limited structural homology. ETV1, ETV4 and ETV5 are the members of a small subfamily of structurally related ETS proteins. These proteins contain in the N-terminal region a conserved short acidic transactivation domain (TAD) that is absent in ERG. ETS proteins regulate many target genes that modulate biological processes like cell growth, angiogenesis, migration, proliferation and differentiation. However, which of the many molecular and biological functions of ETS proteins are most important in prostate cancer is not known.

Following *ERG, ETV1* is the most frequently overexpressed ETS gene in prostate cancer (∼10% of the tumors) [10]. The ETV1 protein translated from most fusion transcripts is truncated, lacking the 131 N-terminal amino acids (dETV1). Approx. half of the tumors with *ETV1* overexpression express a fusion transcript, the others show a high level of full-length *ETV1* expression. The *in vitro* biological and molecular properties of dETV1 seem different from those of the full-length 477 amino acid protein [10]. This observation suggests that tumors with overexpression of full-length ETV1 are different from tumors expressing dETV1.

Little is known about the mechanism of full-length *ETV1* overexpression and its function in clinical prostate cancer. Our present results show that overexpression of full-length *ETV1* is correlated with rearrangement of the *ETV1* chromosomal region. Moreover, we identified a novel, N-truncated *ETV1* isoform with the same activity as full-length *ETV1*.

## Results and Discussion

**Figure 1:**
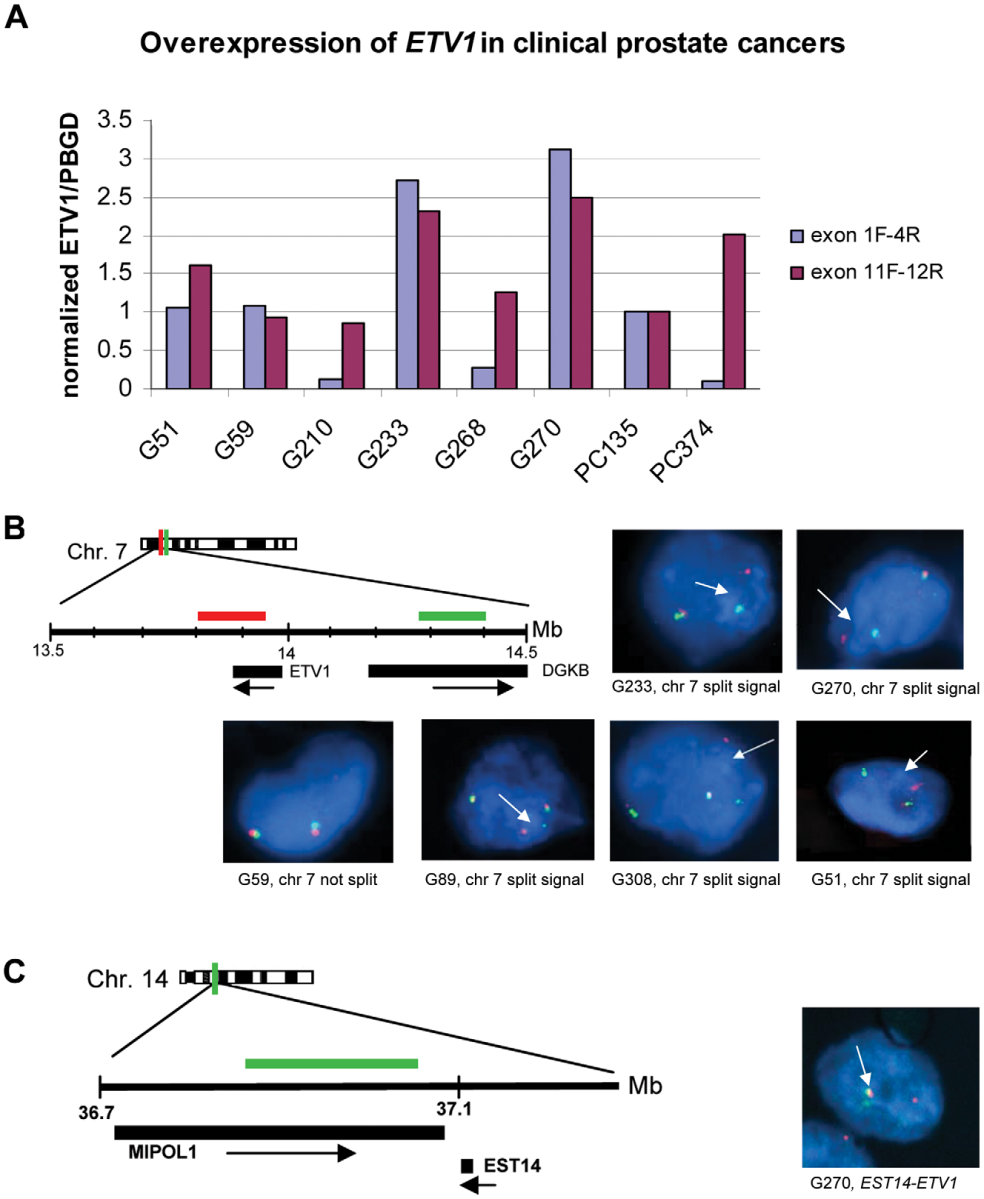
Full-length *ETV1* overexpression in clinical prostate cancers correlates with genomic rearrangement of the complete gene. (A) QPCR on RNA from clinical samples that show *ETV1* overexpression. Two primer-pairs were used to determine *ETV1* expression: *ETV1* exon 1 forward and exon 4 reverse, and exon 11 forward and exon 12 reverse, respectively. Primer sequences are given in Supplementary Table S1. Amplified products were quantified relative to the expression of the porphobilinogen deaminase (*PBGD*) housekeeping gene. Data were normalized to full length *ETV1* overexpressed in the xenograft PC135. A high exon 11/12 to exon 1/4 ratio indicates an *ETV1* fusion event, a 1 to 1 ratio indicates overexpression of a full length *ETV1* transcript. PC374 is a control xenograft that expresses a *TMPRSS2-ETV1* fusion gene. A representative experiment of the six samples that show *ETV1* overexpression is depicted. (B) Interphase FISH on fresh-frozen prostate cancer tissue sections. BACs used are indicated below the chromosome 7 region investigated. BAC RP11-124L22 (red) spans *ETV1* and RP11-1149J13 (green) overlaps *DGKB* (left panel). Positions of genes from the top of chromosome 7 are indicated in Mbp. A split signal representing an *ETV1* translocation is indicated by an arrow. (C) Translocation of *ETV1* to chromosome 14 in tumor G270. Tissue sections were hybridized with BAC RP11-460G19 (green) that overlaps *MIPOL1* and flanks *ETS14* and with the *ETV1* BAC RP11-124L22 (red) (see B for details). In the left panel the position of *MIPOL1* BAC on chromosome 14 is indicated. In the right panel a merging signal (yellow) shows co-localization of *ETV1* and *MIPOL1*/*EST14* in G270, as indicated by the arrow.

Previously, we reported *ETV1* overexpression in eight out of 84 prostate cancer samples. In four cases this was caused by a gene fusion, in the other four a full-length *ETV1* transcript was overexpressed [10]. In the present study we investigated overexpression of *ETV1* by quantitative reverse transcriptase reaction (QPCR) in a novel cohort of 66 prostate cancers. In six RNAs *ETV1* overexpression was detected. Samples G51, G59, G233, G270 and G268 were derived from primary tumors and G210 was derived from a recurrent tumor. The six samples were further studied by QPCR with primer pairs at the 5′-end of *ETV1* mRNA (exon 1F and exon 4R) and at the 3′-end of the mRNA (exon 11F and exon 12R) (Figure 1A). A high exon 11–12 to exon 1–4 ratio is indicative for a fusion gene; a 1∶1 ratio indicated overexpression of full-length *ETV1* mRNA. Based on these criteria, tumors G51, G59, G233 and G270 expressed full-length *ETV1* whereas G210 and G268 expressed a fusion transcript (see also control PC374 that expresses *TMPRSS2-ETV1*). *HNRPA2B1-ETV1* was found as the fusion transcript in sample G210 (data not shown); the fusion transcript in G268 has not been identified as yet. These two samples were not investigated further in this study.

In the next experiments we focused on the elucidation of the mechanism of overexpression of full-length *ETV1*. Recently, it has been shown that in two prostate cancer cell lines overexpressing full-length *ETV1*, LNCaP and MDA PCa2b, *ETV1* is translocated [8]. We now addressed the question whether in clinical samples translocation of the complete *ETV1* gene might occur. To detect genomic rearrangements tissue slides of all four prostate cancer samples with full-length *ETV1* overexpression were analyzed by break-apart interphase FISH with two labeled BAC probes, one BAC recognized *ETV1* and the second one the flanking gene *DGKB* (Figure 1B). The series was supplemented with two samples harboring full-length *ETV1* overexpression from our previous study, G89 and G308 [10], for which frozen tissues of sufficient morphological quality were available. Figure 1B shows the results of the FISH experiments. Interestingly, we found split signals in all samples except for G59, indicating frequent *ETV1* rearrangements in prostate cancers that overexpress full-length *ETV1*. Absence of a split signal in G59 suggests absence of translocation, although we cannot exclude a breakpoint outside the investigated region. Its observed high frequency indicates genomic rearrangement as an important mechanism of full-length *ETV1* overexpression in clinical prostate cancer. Obviously, the chromosomal region to which *ETV1* is translocated can contribute to elucidation of the mechanism of *ETV1* overexpression. We were able to identify more details of the rearrangements in samples G270 and G89.

**Figure 2:**
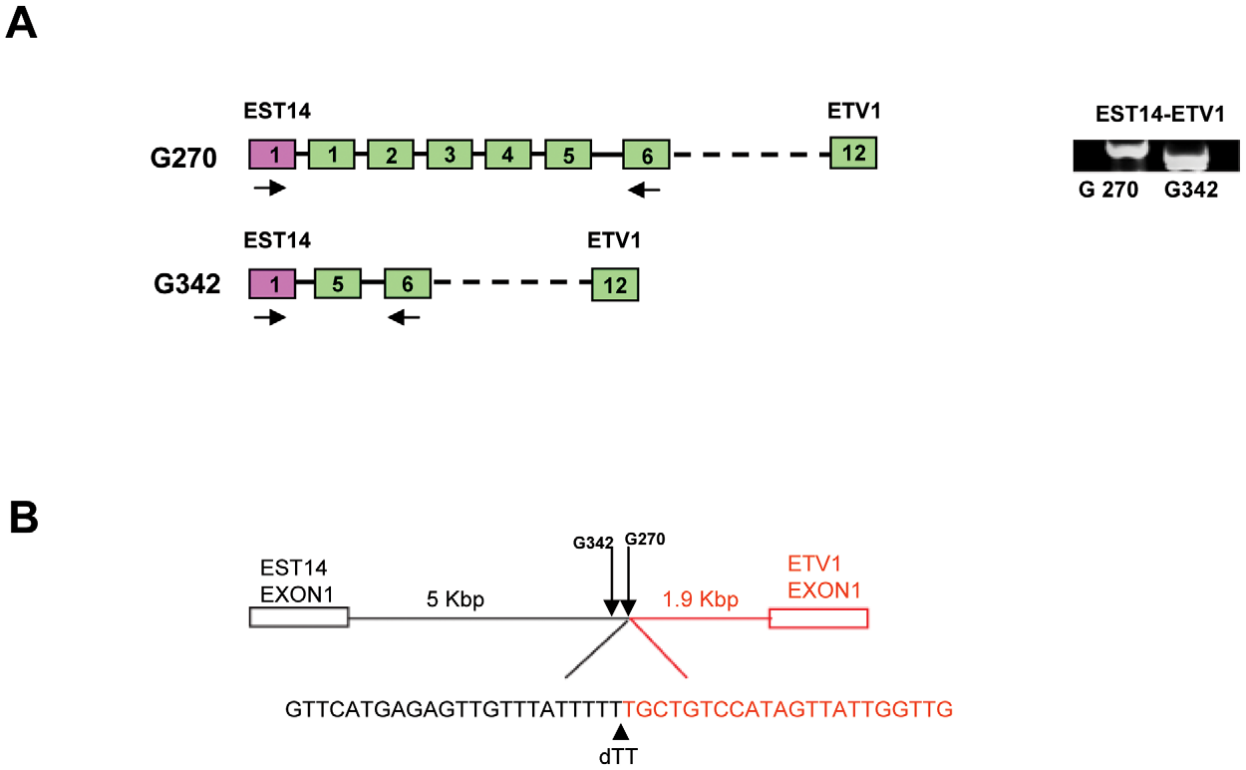
Characterization of the *EST14* to *ETV1* gene fusion in prostate cancer G270. (A) Schematic representation of *ETV1*-*EST14* fusion transcripts in prostate tumors G270 and G342 (sample G342 is from ref. 10). Arrows indicate positions of primers used in the RT-PCR experiment. Primer sequences are shown in Supplementary Table S1. (B) Sequence of the fusion point of *EST14* and *ETV1* in sample G270. The position of the fusion point in tumor G270 was mapped by long-range PCR on genomic DNA with a forward primer in the *EST14* intron and reverse primer upstream of *ETV1* exon 1. At the fusion point two T residues were lost. The breakpoint in *EST14* in G270 and G342 are indicated by arrows.

It has been shown in LNCaP cells that *ETV1* is translocated to 14q13.3-q21.1. The whole gene is integrated in the last intron of *MIPOL1*. In MDA PCa2b, *ETV1* is translocated to the same region, although the precise position is unknown [8]. Moreover, we previously described insertion of truncated *ETV1* in the intron of a two exon gene encoding a ncRNA, denoted *EST14*, giving rise to an *EST14-ETV1* fusion transcript that contains *ETV1* exon 5–12 sequences (sample G342 in ref. 10; Figure 2A). Importantly, *EST14* maps directly adjacent to *MIPOL1* on 14q. Like most *ETV1* fusion partners, *EST14* is an androgen-regulated prostate-specific gene. To investigate whether the same chromosomal region was involved in full-length *ETV1* translocation in our novel cohort, interphase FISH was performed with the *ETV1* BAC (Figure 1B) in combination with a *MIPOL1* BAC (Figure 1C). A merging yellow signal was detected in sample G270 (Figure 1C) but in none of the other tumors. These data indicate that although there seems a preference for chromosome 14q13.3-21.1, other genomic regions will also contribute to rearrangement and overexpression of full-length *ETV1*.

Additional information of *ETV1* rearrangement in G270 came from 5′-RACE of tumor cDNA (data not shown). Remarkably, we did not only detect as expected the full-length *ETV1* transcript but also a fusion transcript (Figure 2A). Such a result was not found for any of the other tumors overexpressing full-length *ETV1*. Sequencing showed that the fusion transcript in G270 was composed of *ETV1* exon 1–12 preceded by the first exon of *EST14* (Figure 2A). Scanning the *EST14* intron and *ETV1* flanking region by long-range PCR and sequencing mapped the breakpoints in G270 ∼1.9 Kbp upstream of *ETV1* exon 1 and ∼5 Kbp downstream of *EST14* exon 1. The breakpoint in *EST14* is only 180 bp apart from the breakpoint in G342 (Figure 2B and ref. 10).

**Figure 3:**
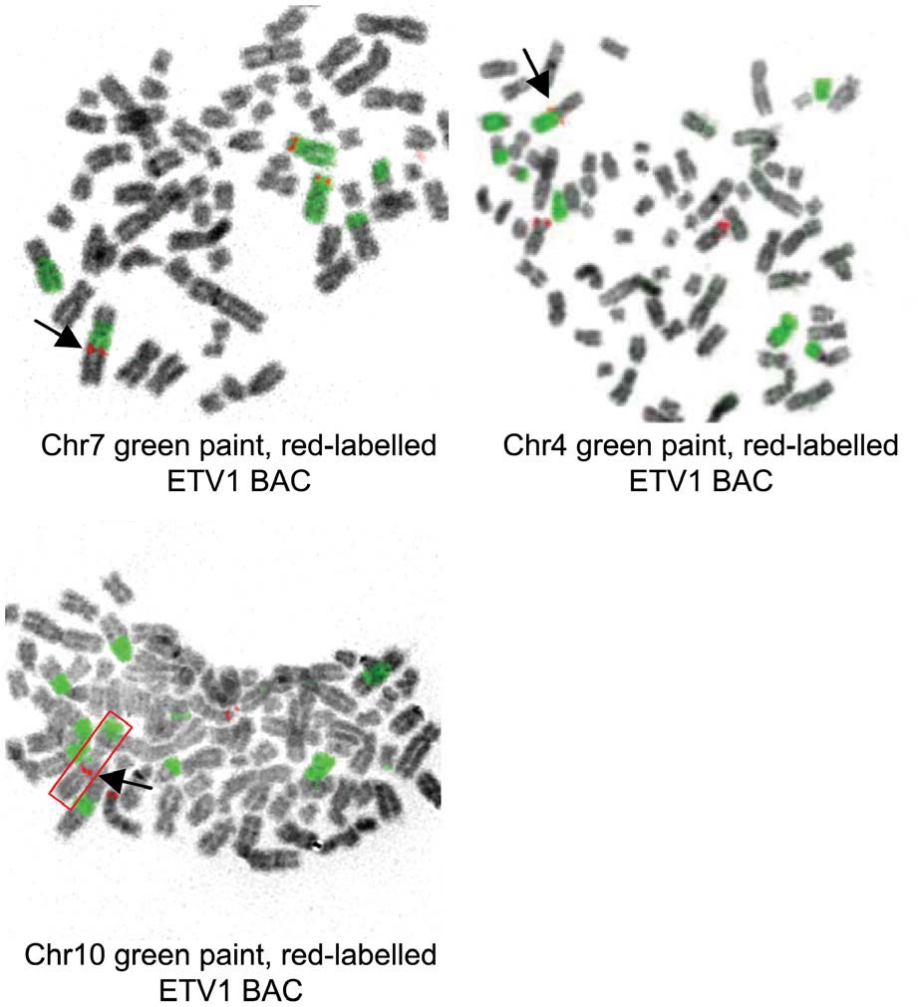
A complex *ETV1* translocation in xenograft PC135 involves chromosomes 4, 7 and 10. Paints of chromosomes 4, 7 and 10 are green and the *ETV1* BAC (Figure 1b) is red. Black arrows indicate the translocated *ETV1*. In the lower panel the relevant marker chromosome is boxed in red.

Further information of *ETV1* rearrangement was also collected for sample G89. Previously, a xenograft propagated on male nude mice had been generated from this tumor (PC135). Like tumor G89, PC135 overexpressed full-length *ETV1* [3]. The availability of the xenograft allowed the preparation of metaphase chromosome spreads. In multicolor FISH a complex chromosomal rearrangement pattern was found (data not shown). Individual chromosome paints were used to validate the multicolor FISH data. Painting of chromosome 7 indicated the presence of multiple chromosome 7 fragments (Figure 3). Hybridization with an *ETV1* BAC identified the presence of three gene copies: two in apparently normal chromosomes 7 and one in a complex marker chromosome. Follow-up experiments showed that the marker chromosome contained fragments of chromosomes 4, 7 and 10, as first indicated by multicolor FISH (Figure 3). The *ETV1* BAC hybridized at the junction of the chromosome 7 and the chromosome 4 fragment, strongly suggesting that the 4;7 translocation was instrumental in overexpression of *ETV1*. The precise positions of the breakpoints in 4 and 7 remain to be determined. Our data predict that multiple chromosomal regions are involved in overexpression of full-length *ETV1*. At least one of these regions is on chromosome 14 and a second one on chromosome 4. The chromosome 14 region is also involved in *ETV1* gene fusion. Deep sequencing technology could be instrumental in identification of other *ETV1* rearrangements.

**Figure 4:**
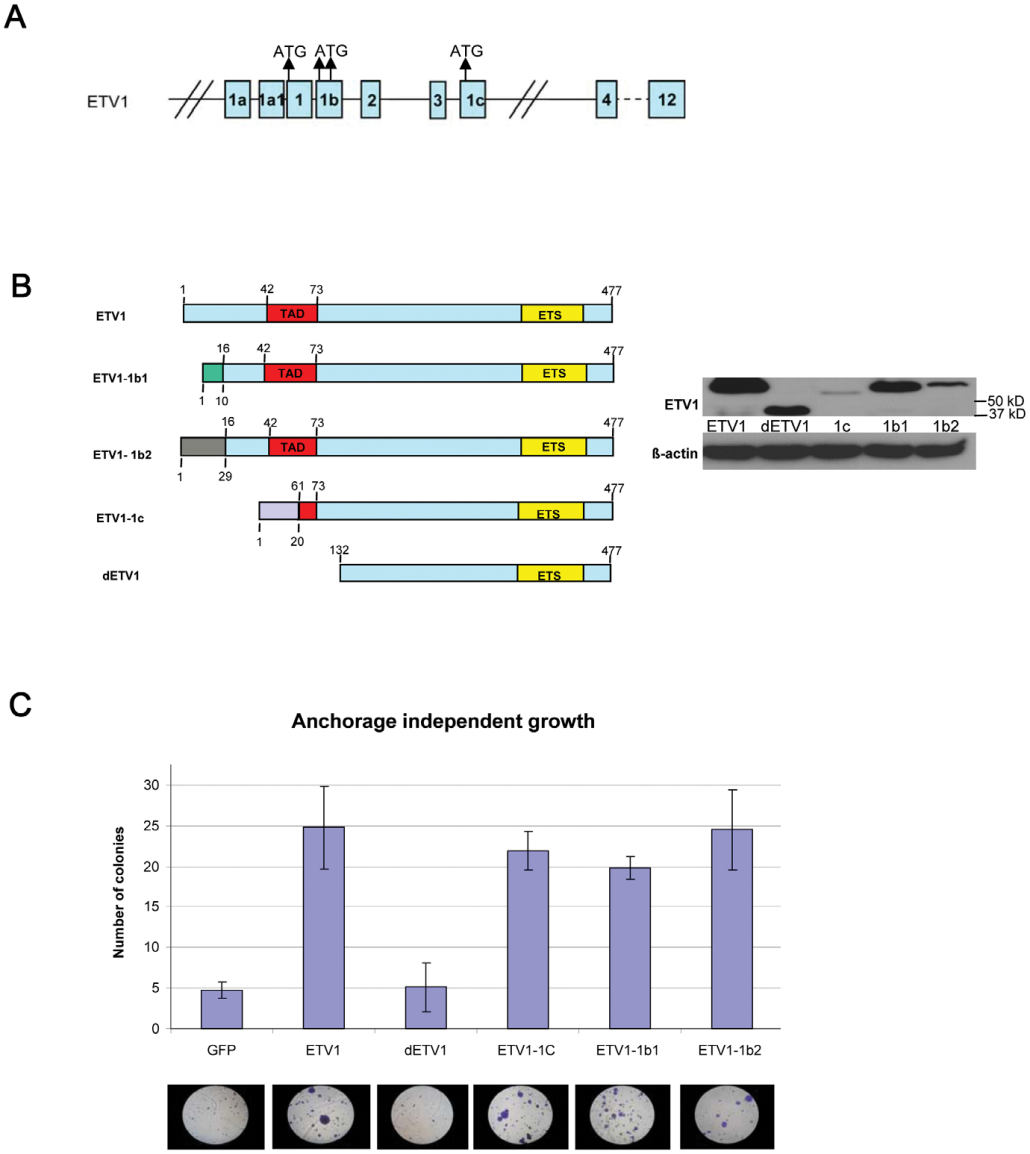
Alternative full-length *ETV1* transcripts give rise to proteins that all induce *in vitro* anchorage-independent growth. (A) Schematic representation of the organization of the *ETV1* gene. Alternative first exons are 1a, 1, 1b and 1c. The positions of the four different ATG start codons are indicated. More details are given in Supplementary Figure S1. (B) Schematic representation of the composition of different ETV1 proteins and their expression in transfected HEK293T cells. Different colors of N-terminal regions indicate a different amino acid composition. dETV1 is the truncated ETV1 protein produced by fusion genes. This protein is unable to induce anchorage independent growth. In the right panel a Western blot of ETV1 isoforms produced by transiently transfected HEK293T cells is shown. β-actin was used as loading control. (C) Soft-agar assay showing the anchorage-independent growth of PNT2C2 cells infected with lentiviruses expressing the various ETV1 isoforms or control GFP. The bars represent the average number of colonies per microscope field of three independent experiments (±SD). Representative images of the stained colonies are shown below the bar figure.

Detailed characterization of the full-length *ETV1* transcripts in the various tumors by 5′-RACE and sequencing showed that not only *ETV1* exon 1- exon 12 transcripts were present but also various other full-length *ETV1* transcripts, resulting from alternative promoter usage. These transcripts were denoted as *ETV1*, *ETV1-1a*, *ETV1-1b* and *ETV1-1c.* Figure 4A and Supplementary Figure S1 show the positions of the different first exons in the gene and indicate the various ATG start codons. Of both *ETV1-1a* and *ETV1-1b* two splice variants were found (data not shown). QPCR experiments using transcript-specific primers on RNA from all six clinical prostate cancer samples that overexpressed *ETV1* showed that the level of expression of the different transcripts was variable in the various tumors (see Figure S2). *ETV1* was hardly expressed in control benign prostate hyperplasia sample G277. Figure 4B schematically represents the predicted composition of the various ETV1 protein isoforms that will be produced. Note that ETV1-1c is by far the shortest, lacking the N-terminal 60 amino acids, including the major part of the conserved acidic TAD. In dETV1 that is expressed by most fusion genes, the N-termimal 131 amino acids are absent. ETV1, ETV1-1b1 and -1b2 were of similar size, as shown in Western blots of lysates from transfected HEK293T cells (Figure 4B).

Previously, we have shown that ETV1 and dETV1 differed in stimulation of *in vitro* anchorage-independent growth [10]. PNT2C2 cells infected with all novel ETV1 constructs induced anchorage-independent growth in a similar manner as ETV1 (Figure 4C). Remarkably, ETV1-1c, although expressed at a lower level and much smaller, is as active as the longer ETV1 isoforms. Thus, the full N-terminal TAD was not needed but amino acids 61–131 seem essential for biological activity of ETV1 (Figure 4B, C).

In summary, the data presented reveal two important novel aspects of the role of ETV1 in prostate cancer. First, it is shown that in clinical prostate cancers a subgroup of ETV1 positive patients show full-length *ETV1* overexpression due to translocations of the whole gene to different chromosomes. This novel observation complements the well-described mechanism of overexpression of truncated ETV1 caused by gene fusions where expression regulation is determined by the promoter and enhancers of the fusion partners. Secondly, in contrast to dETV1 produced by gene fusions, a short isoform of full-length ETV1, ETV1-1c, lacking most of the N-terminal TAD, is as active as longer ETV1 isoforms, containing the complete N-terminal acidic TAD. This finding pinpoints the anchorage-independent growth to a small region that is absent in truncated ETV1 expressed by fusion genes.

It is highly relevant to extend the number of clinical samples in order to be able to compare tumor progression in the two subgroups of prostate cancers showing overexpression of truncated vs. full-length ETV1, and to determine the molecular mechanisms involved in their different biological behavior.

## Materials and Methods

### Ethics Statement

Use of the samples was approved by the Erasmus MC Medical Ethics Committee according to the Medical Research Involving Human Subjects Act in protocol MEC-2004-261, entitled “The use of human normal and cancer residual tissue from a tissue bank for characterization of DNA, RNA and protein”.

Mice were housed according to guidelines of the Erasmus Medical Centre, and procedures were carried out in compliance with standards for use of laboratory animals. Animal experiments performed in this manuscript have been approved by the animal experimental committee of the Erasmus Medical Centre (DEC-consult Erasmus MC project 102-10-01).

### Tissue samples, RNA and DNA isolation

Snap-frozen prostate cancers were obtained by radical prostatectomy or transurethral resection. Hematoxilin/eosin (HE) stained tissue sections were histologically evaluated by a pathologist (G. van Leenders). All samples contained at least 50% tumor cells.

RNA from clinical specimens was isolated using RNA-Bee (Campro Scientific, Berlin, Germany). DNA was isolated using the DNeasy DNA Extraction kit (Qiagen, Valencia, CA, USA). RNA from cell lines was isolated using the RNeasy RNA Extraction kit (Qiagen).

### Breakpoint mapping

The position of the fusion point in tumor 270 was mapped by long-range PCR on genomic DNA using a forward primer in the *EST14* intron and reverse primer upstream of *ETV1* exon 1. PCR products were separated on a 1% agarose gel and sequenced in an ABI 3100 genetic analyzer (Applied Biosystems, Carlsbad, CA, USA).

### Q-PCR

mRNA expression was analyzed by QPCR. cDNA was prepared with MMLV-RT (Invitrogen, Carlsbad, CA, USA) and oligo(dT)12 primer. QPCR was performed in Power SYBR Green PCR Master Mix on an ABI Prism 7700 Sequence Detection System (Applied Biosystems). Amplified products were quantified relative to porphobilinogen deaminase (*PBGD*) by the standard curve method. For primers see Table S1.

### RNA ligase-mediated rapid amplification of cDNA ends

5′-RLM-RACE was performed using the GeneRacer kit of Invitrogen. cDNA was amplified using the GeneRacer 5′-primer and a reverse gene-specific primer (ETV1 exon 6). PCR products were analyzed on a 1.5% agarose gel, and bands were excised, purified, and sequenced.

### Fluorescence *in situ* hybridization

FISH was done on 5-µm frozen tissue sections according to standard protocols with minor modifications [14]. BAC clones RP11-124L22 (*ETV1*), RP11-460G19 (*MIPOL1*), RP11-1149J13 (*DGKB*) were purchased from BacPac Resources (bacpac.chori.org). BACs were either digoxigenin-11-dUTP or biotin-16-dUTP (Roche, Basel, Schweiz) labeled and visualized with anti-digoxigenin FITC (Roche) or streptavidin-Alexa 594 (Invitrogen). Tissue sections were counterstained with DAPI. Images were collected on an epifluorescence microscope (Leica DM, Wetzlar, Germany) equipped with a charge-coupled device cooled camera (Photometrics, Tuscon, AZ, USA).

For preparation of metaphase spreads, xenograft PC135 was propagated on male nude mice. Single cells were collected by mincing and filtration. Metaphase preparation and hybridization were essentially as described [14]. Chromosome paints 4, 7 and 10 were from Euro-Diagnostica (Malmö, Sweden). Metaphases were analyzed with an Axioplan 2 Imaging microscope (Carl Zeiss, Oberkochen, Germany) and images were captured using Isis software (MetaSystems, Altiussheim, Germany).

### Expression plasmids

cDNAs of the different ETV1 isoforms were PCR amplified and cloned into pGEM-TEasy (Promega). Inserts were sequence verified and cloned into the pcDNA3 expression vector (Invitrogen) or the lenti-viral vector pWPXLd (Didier Trono, University of Geneva).

### Western blot analysis

For Western blot analysis, HEK293T cells were transfected with the different pcDNA3-ETV1 expression constructs using the calcium phosphate precipitation method. Cells were harvested 48 h after transfection. Western blot analysis was carried out using standard procedures with antibody directed to the ETV1 C-terminus (Santa Cruz, Santa Cruz, CA, USA). β-actin was used as loading control (Sigma, St Louis, MO, USA). Proteins were visualized by chemiluminescence (Pierce, Rockford, IL, USA).

### Lentiviral infections

HEK293T cells were cotransfected with pWPXLd-ETV1 expression vectors, or pWPXLd-GFP (control), and pPAX2 and pMD2.G (Trono) using the calcium phosphate precipitation method. Virus was harvested from the supernatant and used for infection of PNT2C2 cells. Pools of infected cells were propagated.

### Soft agar assay

A layer of 0.6% low-melting agarose in standard culture medium was prepared in six-well plates. On top, a layer of 0.3% agarose containing 1×10^4^ PNT2C2 cells infected with various ETV1 expressing viruses or control PNT2C2-GFP cells were plated. At day 14, cells were stained with crystal violet and colonies were counted.

## Supporting Information

Figure S1 Part of genomic sequence of *ETV1*. The different exons are highlighted in yellow. Translation start codons are underlined. Note that the transcript starting at exon 1a may or may not include exon 1a1 but the translation start is the same as that of the transcript starting at exon 1. The end of exon 1b1 is indicated in red. *ETV1* transcripts starting at exon 1c lack exons 1, 2 and 3 which results in a truncated TAD in the translated protein.
(DOC)

Figure S2 Expression of the different *ETV1* transcripts was determined by QPCR in the 7900HT Fast Real-Time PCR system from Applied Biosystems using the power SYBR-green master mix (Applied Biosystems). Expression levels are relative to the housekeeping gene *PBGD*. *ETV1* and *PBGD* primers are listed in Supplementary Table S1. Sample G277 is a BPH. It has very low or no expression of all of the different *ETV1* transcripts. Samples G51, G59, G89, G233, G270 and G308 all overexpress *ETV1.* The different transcripts are expressed in variable levels.
(TIF)

Table S1 Primer sequences.
(TIF)

## References

[1] Futreal PA, Coin L, Marshall M, Down T, Hubbard T (2004). A census of human cancer genes.. Nat Rev Cancer.

[2] Tomlins SA, Rhodes DR, Perner S, Dhanasekaran SM, Mehra R (2005). Recurrent fusion of TMPRSS2 and ETS transcription factor genes in prostate cancer.. Science.

[3] Hermans KG, van Marion R, van Dekken H, Jenster G, van Weerden WM (2006). TMPRSS2:ERG fusion by translocation or interstitial deletion is highly relevant in androgen-dependent prostate cancer, but is bypassed in late-stage androgen receptor-negative prostate cancer.. Cancer Res.

[4] Perner S, Demichelis F, Beroukhim R, Schmidt FH, Mosquera JM (2006). TMPRSS2-ERG fusion-associated deletions provide insight into the heterogeneity of prostate cancer.. Canser Res.

[5] Wang J, Cai Y, Ren C, Ittmann M (2006). Expression of variant TMPRSS2/ERG fusion messenger RNAs is associated with aggressive prostate cancer.. Cancer Res.

[6] Tomlins SA, Mehra R, Rhodes DR, Smith LR, Roulstron D (2006). TMPRSS2:ETV4 gene fusions define a third molecular subtype of prostate cancer.. Cancer Res.

[7] Helgeson BE, Tomlins SA, Shah N, Laxman B, Cao Q (2008). Characterization of TMPRSS2:ETV5 and SLC45A3:ETV5 gene fusions in prostate cancer.. Cancer Res.

[8] Tomlins SA, Laxman B, Dhanasekaran SM, Helgeson BE, Cao X (2007). Distinct classes of chromosomal rearrangements create oncogenic ETS gene fusions in prostate cancer.. Nature.

[9] Attard G, Clark J, Ambroisine L, Mills IG, Fisher G (2008). Heterogeneity and clinical significance of ETV1 translocations in human prostate cancer.. Br J Cancer.

[10] Hermans KG, van der Korput HA, van Marion R, van de Wijngaart DS, Ziel-van der Made A (2008). Truncated ETV1, fused to novel tissue-specific genes, and full-length ETV1 in prostate cancer.. Cancer Res.

[11] Wasylyk B, Hahn SL, Giovane A (1993). The Ets family of transcription factors.. Eur J Biochem.

[12] Oikawa T, Yamada T (2003). Molecular biology of the Ets family of transcription factors.. Gene.

[13] Seth A, Watson DK (2005). ETS transcription factors and their emerging roles in human cancer.. Eur J Cancer.

[14] Eussen BH, van de Laar I, Douben H, van Kempen L, Hochstenbach R (2007). A familial inverted duplication 2q33-q34 identified and delinieated by multiple cytogenetic techniques. Eur J Med.. Genet.

