# Supplementary material for: Overexpression of Full-Length ETV1 Transcripts in Clinical Prostate Cancer Due to Gene Translocation
Source: PLoS One. 2011 Jan 26;6(1):e16332. doi: 10.1371/journal.pone.0016332 (PMC3027657; doi:10.1371/journal.pone.0016332)
Supplement: Figure S1 — Part of genomic sequence of ETV1. The different exons are highlighted in yellow. Translation start codons are underlined. Note that the transcript starting at exon 1a may or may not include exon 1a1 but the translation start is the same as that of the transcript starting at exon 1. The end of exon 1b1 is indicated in red. ETV1 transcripts starting at exon 1c lack exons 1, 2 and 3 which results in a truncated TAD in the translated protein. (DOC) [file pone.0016332.s001.doc]

AGTAGAGGAA AAACCACCGA ATTGAGCTCT GTCAGAGGCG CTTTCGGCTT CCAAGGGGGA

AGTGCTGGGC TATAATTAAT GTTTTTATTA AATTTGGAGG GAAGTTTTTG CAGCCTTTCG Exon 1a

CCTAGCGTGG CCTTCAGGTG GGTCTTTCCA ACAACTTTTT ATGTCTCTCC AGATAATTGC

ATGGTTGTGG GTCGCAAAAA CTATCCTGAT GAAAGAGGTG TCTCCGTCTC TTTAGTCCCG

TTAGGTGCAA AGCAAGTCTC GTTGATCGCC ATTGCTAGTT TTGCACACGT TTGCGAATCA

GAGCTGCCCG GGGTACACCG ACCGCGCAGG GAAACATCGA GAGTGTAAAT AAATACATCG Exon 1a1

CCTCTTGTTC GGATTTTTGC TACTACCGAA AATATGTAAA TTGTGAACTC TGTTGGCTCT

CTTTGGATCC AGGTGAAGGA GGCGGTGTAG GGAGGGTTAT TTTTGTGAAT GGGACTGTCG

GAGGGTAATT AGCTATGCAA ATTCAAGAGC TTCATTCATG ATTTTTTTTT AAGCCTAAAA

GCCATCTTGT TCCCCTCTAG GTTGATAGAA GTCCAGATCC TGAGGAAATC TCCAGCTAAA

TGCTCAAAAT ATAAAATACT GAGCTGAGAT TTGCGAAGAG CAGCAGCATG GATGGATTTT Exon 1

ATGACCAGCA AGTGCCTTAC ATGGTCACCA ATGTGAGTGA TCAGTTTGAA AGTTGCTGTT

TATAAACTTG ACTCCGTCGG GGGTGGGGAG AGGGAGAAAA TGAGAAGGGA GGGGGCACGG

GGGTTGGGAT TGCAGATACT TATCTGCTTT GTTGCCACTG TAGGGCGACT CTGCTTCTAG

AAGCCCAGTC TTCAAAATGA GCTTACCTTT CAGTGATTTG GATAAGGCAT AGTTTTGTTT Exon 1b1/1b2

TTAAGACCCC TTTTTCTGAT TAAAGTGCCC AACATGAGTG GAAGAGGAGA TGGAGGGCAG

CAGCAGCTGC TGCACTCAAA GTTTTTGGCT GGGTTTGTCT GCCACATTGA AAAGAATGAA

GTTGAGACAA ATGCTGACAC TTTTTGTTTA TATGGGGTGT TTTTGTTTCT TTTTTTTTTC

TCATTTTCTC TTTTTTTTTT TTTTCATTTC TCTACTCTTT TTGCTTTTTT TTTTTGTGGG

GGTGGGGTGG GGAGGAGGGG CTGTCCAAGA GAGAATAAAA CCCACTGTTT TAATCTAGCA

TTGAACCAGC CTAAAAACAA CTTTAAGTGT AAATGCTTCA GGTTGTTTTG ATGCTGAGAT

ACCATGAATA TATGATTGAT AAATAGTAAT GTGCTAAGAT CGGCACTGGG AAGCAACTCT

CCCTGCCTGC TTAACATAAG CTTTCACTGT CAAATAAGTG AGAAAATGAA TATTAGAGTT

GATACTATGG ACTTCTATTT TTCTATTCAG AGAAGATCCA TTAGACTTAT GTATGCATGT

CTGTGTGTGT GTGTGTGCGC GCGCGTGTGC GTGTGTGTGA TCTGAGGTTT ACATTCTTTT

AAAGGATTTT ATCTTTCCCT TTGTAGAGTC AGCGTGGGAG AAATTGTAAC GAGAAACCAA Exon 2

CAAATGTCAG GAAAAGAAAA TTCATTAACA GAGATCTGGC TCATGATTCA GAAGGTGAGG

TTTGATTTTG GGCTGAATCC CCATTTTTTT ACCTACACTC TCCACCCTGC TAAAAGAAAA

GGAGCACTTC AGTCTTATCT TAAAAATAAT AAACTTTGAA ATTACTGAAT CTAACTAAGA

CTTTAACTTG GAACGATTTA CATAATGCGG CAGTTTGCAT GGGAGACTAT TTTATGGTCC

TATCACAAAG TAATGAAAGA AGGAAATAAA AACTTCTCAA TGACAACCTT CTTTTAATCA

TTGTTTTGTG TTTAAATACC TTTGTCCATG GGGCATATAA CTCACCCCAT TCACTAAGAT

AACAATTTAA AAATATCCAA TACAGTGGGA CAAATTCAGC AGCTATGTAC ACTGCTTACT

TTTTTCTTTC TCCTTTATTA CAAGTTAAAC AGTATTAGTG ATTGTTTGGT TTCCCACCTC

CTTTACACTA AAACCATCTA CTATTAACAG AATTTTCAAG GGTGATTTTG ATGACTAAAT

TGTGAGGTCA TAATACCTTG TTTATCGACC ATAATGTGTT CACTGAATCA TACTTAAGGC

CTCCTTAATA ATTATAATAC TATTTGGGAA ATTAGTTTGC AGATTCTTTT TATGAACTGT

TGCTAGGATG TCAGTATGTT ACAAATTATT TTAGCCATAT GAGGACATTC ATAATAAACT

TAATATACTT AGATTTAATT ATCAAAGTTT AGTTGGCAAT TTTAATTAGA TATACTGAAT

TCTGAAACTT CCTCAAATCT TCATACCATT TAAATACCTC CCTCTCTCCC TCCCTAACAT

GACCATATAG TTTAGAACTT GATGAGAACC TGGTGCCAAA GAATTATCTT TCTGACTTTT

ATAAACCTAT TAATGTAGAA ATTTTCCTCT AAAAACTTGT ATTTGAAGTG GTTTCTTTTA

GTTTCGAGGT ATTGAAGTTG AAAACATAAT ACTTTGACAG TTGTACCATG TACCATTATA

TATTTTATGT AAGATTCAGA AGATATAAAT AAAATGCTTT TCAGTCTTTC TAATCTTCCC

CCTACTACTA TTTTAGCAAG AGTTAAATAT ATTTAAGGAA GATTTCAGTG AACAGACACA

TATTCACCTC AGCCTTTTTG TGAATTCTGG TATCCCTGAG CTTATTTATT AAGCTGTTTG

TTAAAAAGAA GAAAAGATGA TTGACTTTAT TCTTTAGTTG ACTAAAATAA TTTCTTAAAG

AGTTGCTTAA TAGGCATGAA AAGTAGGCAG TATTATTGAG ATATTTAAGT TATGCCTCTT

TTTTTTATTT TTATGTTGAC TGCGTCTCTT GAAATTAAAT TTATATTTAA TACCAATTTC

ATGACAAATA TTTCCATTTA ATGAAGATTA AAGATTTGCA AATCTTATGT TTATGTCTTT

GACTTGTTTT TTAGAACTCT TTCAAGATCT AAGTCAATTA CAGGAAACAT GGCTTGCAGA

AGGTAAGGCA AAATTTGCTT TAAAAGGGGG GAAAGCAACT CTAGAAGGAG AAAGAAAAGA Exon 3

AGTCCTGAAC TTGCTGTCTT AATATTCAGC CCAATTAATT GAGCTCTAAA AGAGCCACCT

CATGTGTCAT GCATACATTA GAGCCTCTGA TGAGTTTGTC TTTGGGGACT CTGGGGCTGT

ACTACCCAGT GTCATCACAA ATTACCAGGA GAAATTGCTT CCAGCTCACA ATCACATCTG

CTTTTGGCAA GAACTAATGC ACCAAGACTT CAAGTTCTAA GCCTCTGTTC AGATTTTAAT

TGCAATTGAT CAGGTTTATA TTATTGTACC TCCAGAGACC TCCTAGAGCC AGAACCCGGC

TGGCTTGCTG TTTCCTTTAG AGCAGCGCAT ATCATTATTT GGTGTTCTGG TGGAGGACTT

TTCTGATGGC AGAAATTAGT TTCTCTGGGT TCATCAGGAC GGGATGCTTC AAGATTTAAG Exon 1c

TGCAAGTGTC TTCTTTCCAC CTTGTTCACA ACACAGAACG TTAGGTGTGT ATCTAATGCT

TAGGAAATCT

Supplementary Figure 1, Gasi et al.
