# Supplementary figures and images for: Overexpression of Full-Length ETV1 Transcripts in Clinical Prostate Cancer Due to Gene Translocation
Source: PLoS One. 2011 Jan 26;6(1):e16332. doi: 10.1371/journal.pone.0016332 (PMC3027657; doi:10.1371/journal.pone.0016332)

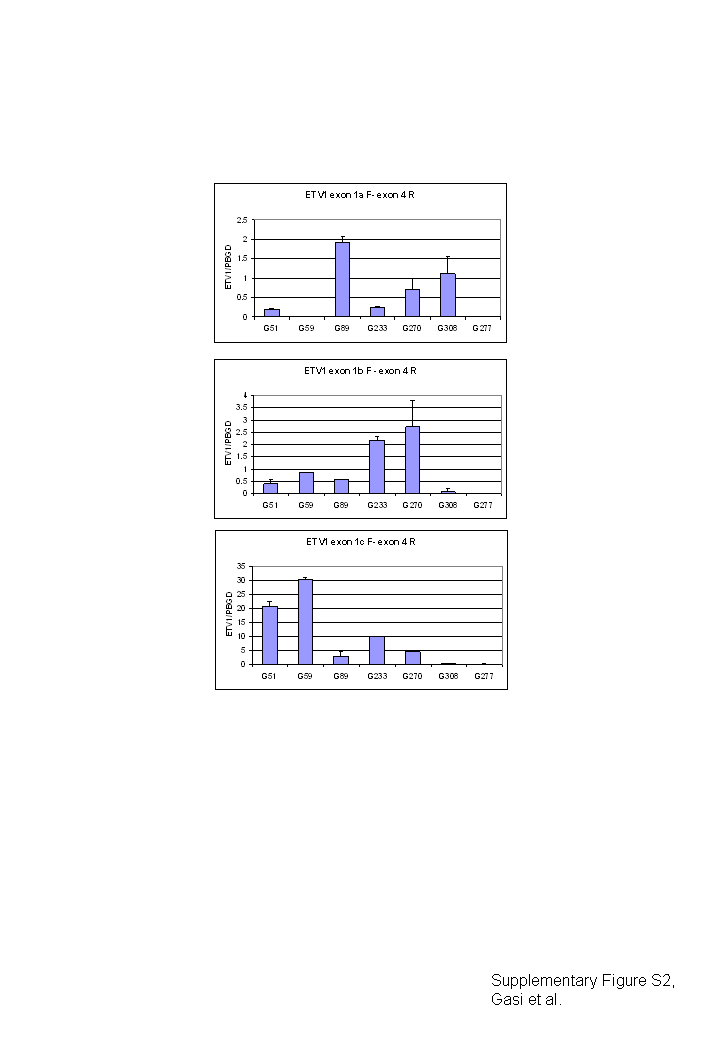

Supplement: Figure S2 — Expression of the different ETV1 transcripts was determined by QPCR in the 7900HT Fast Real-Time PCR system from Applied Biosystems using the power SYBR-green master mix (Applied Biosystems). Expression levels are relative to the housekeeping gene PBGD. ETV1 and PBGD primers are listed in Supplementary Table S1. Sample G277 is a BPH. It has very low or no expression of all of the different ETV1 transcripts. Samples G51, G59, G89, G233, G270 and G308 all overexpress ETV1. The different transcripts are expressed in variable levels. (TIF) [file pone.0016332.s002.tif]

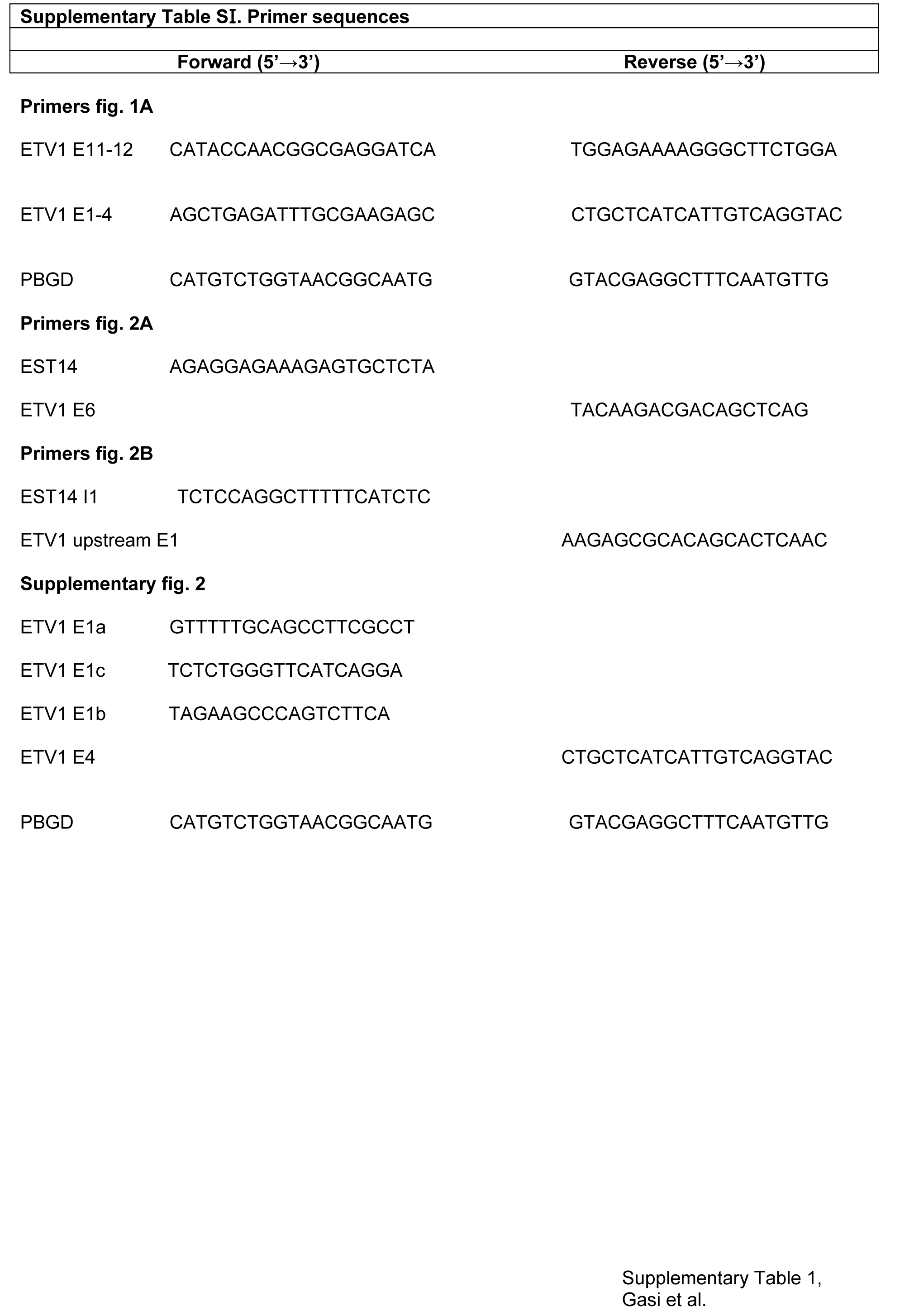

Supplement: Table S1 — Primer sequences. (TIF) [file pone.0016332.s003.tif]
